# Chicken innate immune response to oral infection with *Salmonella enterica* serovar Enteritidis

**DOI:** 10.1186/1297-9716-44-37

**Published:** 2013-05-20

**Authors:** Marta Matulova, Karolina Varmuzova, Frantisek Sisak, Hana Havlickova, Vladimir Babak, Karel Stejskal, Zbynek Zdrahal, Ivan Rychlik

**Affiliations:** 1 Veterinary Research Institute, Hudcova 70, Brno 621 00, Czech Republic; 2 Core Facility - Proteomics, Central European Institute of Technology, Masaryk University, Kamenice 5, Brno 625 00, Czech Republic

## Abstract

The characterization of the immune response of chickens to *Salmonella* infection is usually limited to the quantification of expression of genes coding for cytokines, chemokines or antimicrobial peptides. However, processes occurring in the cecum of infected chickens are likely to be much more diverse. In this study we have therefore characterized the transcriptome and proteome in the chicken cecum after infection with *Salmonella* Enteritidis. Using a combination of 454 pyrosequencing, protein mass spectrometry and quantitative real-time PCR, we identified 48 down- and 56 up-regulated chicken genes after *Salmonella* Enteritidis infection. The most inducible gene was that coding for MMP7, exhibiting a 5952 fold induction 9 days post-infection. An induction of greater than 100 fold was observed for IgG, IRG1, SAA, ExFABP, IL-22, TRAP6, MRP126, IFNγ, iNOS, ES1, IL-1β, LYG2, IFIT5, IL-17, AVD, AH221 and SERPIN B. Since prostaglandin D2 synthase was upregulated and degrading hydroxyprostaglandin dehydrogenase was downregulated after the infection, prostaglandin must accumulate in the cecum of chickens infected with *Salmonella* Enteritidis. Finally, above mentioned signaling was dependent on the presence of a SPI1-encoded type III secretion system in *Salmonella* Enteritidis. The inflammation lasted for 2 weeks after which time the expression of the “inflammatory” genes returned back to basal levels and, instead, the expression of IgA and IgG increased. This points to an important role for immunoglobulins in the restoration of homeostasis in the cecum after infection.

## Introduction

*Salmonella enterica* is one of the most frequent causative agents of human gastrointestinal disorders with the major sources of *S*. *enterica* isolates for the human population originating from farm animal production, pigs and poultry in particular. Poultry flocks are reservoirs of *S*. *enterica* serovar Enteritidis (*Salmonella* Enteritidis), the serovar whose incidence in the human population has increased considerably since the beginning of the 1990s [1,2]. As poultry is a major source of *Salmonella* Enteritidis for humans, it is believed that the measures applied in chicken egg production, which will lead to a decrease of *Salmonella* Enteritidis prevalence, will also affect the incidence of salmonellosis in the human population. This is why a program aimed at decreasing the prevalence of *Salmonella* in poultry flocks is currently implemented in the EU [1,2].

Despite the absence of gross clinical signs, chickens respond to oral infection with non-typhoid serovars of *S*. *enterica* by moderate inflammation in the cecum associated with heterophil and monocyte/macrophage infiltration into the cecal mucosa. In agreement with this, the expression of proinflammatory cytokines such as IL-1β, IL-6, IL-17, IL-22 and IFNγ together with iNOS is increased in the cecum of infected chickens [3-5]. The production of the above cytokines is either induced in epithelial cells and resident phagocytes, or is affected by infiltrating phagocytes or lymphocytes [6]. However, this is clearly a simplified and an incomplete view of the chicken’s response to *Salmonella* infection as, for example, in mice genes not involved in cytokine signaling, e.g., Lcn2, are induced in the small intestine upon infection with *Salmonella enterica* serovar Typhimurium [7]. Moreover, by using pyrosequencing of RNA transcripts in the spleen of chickens intravenously infected with *Salmonella* Enteritidis, we have identified many new genes which have not been associated with *Salmonella* infection in chickens so far [6]. When we tested whether these genes were also induced in the cecum, 14 of them were indeed upregulated in the cecum of orally infected chickens, one of them being a functional homologue of murine Lcn2 [6].

This prompted us to perform a genome-wide characterization of the chicken response in the cecum to oral infection with *Salmonella* Enteritidis. Unlike our previous paper on expression in the spleen [6], we characterized both the transcriptome and proteome by pyrosequencing and protein mass spectrometry, respectively, and the initial results were verified by real time RT-PCR. The combination of both experimental procedures allowed us to characterize the events occurring in the chicken cecum at quite a detailed level and define the individual steps in the chicken’s innate immune response to oral infection with *Salmonella* Enteritidis.

## Material and methods

### Ethics statement

The handling of animals in the study was performed in accordance with current Czech legislation (Animal protection and welfare Act No. 246/1992 Coll. of the Government of the Czech Republic). The specific experiments were approved by the Ethics Committee of the Veterinary Research Institute (permit number 48/2010) followed by the Committee for Animal Welfare of the Ministry of Agriculture of the Czech Republic (permit number MZe 1226).

### Experimental animals, sample collection and pyrosequencing

The ceca of 6 ISA Brown chickens were used for simultaneous RNA and protein isolation. Three of these chickens were infected orally with 10^7^ CFU of *Salmonella* Enteritidis on the day of hatching and sacrificed 4 days later while the other 3 represented non-inoculated 5-day-old control chickens. Approximately 30 mg of cecum was collected from each chicken during necropsy and immediately placed into RNALater (Qiagen). The samples were then mixed with TRI Reagent and processed according to the manufacturer’s recommendations (MRC). In brief, after centrifugation for 15 min at 14 000 *g*, the upper phase containing RNA was purified with an RNeasy Mini Kit (Qiagen) followed by an mRNA Isolation Kit (Roche) to enrich the RNA preparation for mRNA species. Proteins captured in the lower phase were precipitated from the TRI Reagent with 3 volumes of acetone, washed with guanidine hydrochloride (0.3 M)/ethanol (95%)/glycerol (2.5%) wash solution and dried in a SpeedVac.

Because of the cost of pyrosequencing (see below), only pooled RNA/cDNA samples of 3 infected and 3 non-infected chickens were processed, respectively. For protein mass spectrometry, the samples from the 3 infected and 3 non-infected chickens were randomly grouped into 3 pairs and analyzed.

To verify the results from mRNA pyrosequencing and protein mass spectrometry, cDNA samples kept at −20°C from our previous experiments were used in real-time PCR quantification [6]. These samples originated from the cecum of orally infected 5-day-old chickens and included samples from 2 independent experiments, each performed with 4 infected and 4 non-infected chickens.

### Time-dependent gene expression in chicken cecum after *Salmonella* Enteritidis infection

A time-dependent characterization of gene expression in the cecum was performed with 64 chickens which were orally infected with 10^7^ CFU of *Salmonella* Enteritidis on the day of hatching and sacrificed on days 2, 3, 4, 5, 6, 7, 8, 9, 10, 11, 12, 15, 18, 22, 25 and 29 of life, 4 chickens on each day. On day 1 and at all additional sampling times, 4 non-infected control chickens were sacrificed as well. From each of the chickens, mRNA from cecum was purified, reverse transcribed into cDNA and subjected to real time PCR as described below. To verify the infection, *Salmonella* Enteritidis counts in the liver, spleen and cecum were determined by serial dilutions of tissue homogenates in peptone water and plating on XLD agar, as described previously [8].

### Influence of *Salmonella* Enteritidis SPI1 on the inflammation in chicken cecum

In the last experiment, we infected 8 newly hatched chickens with the wild-type *Salmonella* Enteritidis or an isogenic SPI1 mutant of *Salmonella* Enteritidis [9], and sacrificed them 4 days later. Four non-infected control chickens sacrificed on day 5 of life were included as well. During necropsy, cecal wall samples were placed in RNALater followed by RNA purification using an RNeasy Mini Kit (Qiagen).

### 454 pyrosequencing

cDNA libraries from the cecal samples were prepared with the GS Rapid Library Preparation Kit (Roche) and approx. 2 molecules of cDNA per bead were used in emulsion PCR. All steps of the cDNA library preparation were performed with the GS Junior Titanium series kits according to the manufacturer’s instructions (Roche). The pyrosequencing was performed with the GS Junior 454 sequencer (Roche) and each sample was sequenced in a separate sequencing run.

Data analysis after pyrosequencing was performed exactly as described previously except for the fact that the total chicken transcriptome was generated using the sequences from the previous pyrosequencing of the spleen samples enriched for the two new sequencing runs from the cecum [6]. Transcripts predicted as differentially expressed after *Salmonella* Enteritidis infection included those for which we identified at least a 12-fold up- or down-regulation regardless of the number of reads available, or a 6-fold up- or down-regulation with 25 independent reads available, or a 3-fold up- or down-regulation with at least 50 independent reads.

### Protein identification and quantification

Protein pellets from 3 infected and 3 non-infected chickens were processed by the filter-aided sample preparation (FASP) method [10]. Pellets were diluted in 100 μL of 4% SDS, 0.1M DTT, 0.1M Tris–HCl pH 7.6, incubated for 5 min at 95°C, cooled to room temperature and centrifuged at 20 000 × *g* for 15 min at 20°C. The supernatant was collected and mixed with 8 M UA buffer (8 M urea in 100 mM Tris–HCl, pH 8.5) at a ratio of 1:13, loaded onto the Vivacon 500 device with MWCO 10 kDa (Sartorius Stedim Biotech) and centrifuged at 14 000 × *g* for 30 min at 20°C. The retained proteins were washed with 400 μL UA buffer. The final protein concentrates kept in the Vivacon 500 device were mixed with 100 μL of UA buffer containing 50 mM iodoacetamide and incubated in the dark for 30 min. After an additional centrifugation, the samples were washed three times with 400 μL UA buffer and three times with 200 μL of 50 mM NaHCO_3_. Trypsin (sequencing grade, Promega) was added onto the filter and the mixture was incubated for 2 h at 40°C. The tryptic peptides were finally eluted by centrifugation followed by two additional elutions with 50 μL of 50 mM NaHCO_3_. The concentration of the eluted tryptic peptides was determined by UV-spectrometry using an extinction coefficient of 1.1 for 0.1% (g/L) solution at 280 nm [11].

Tryptic peptides prepared by FASP were labeled as described elsewhere [12]. The concentration of each sample was adjusted with 50 mM NaHCO_3_ to a final concentration of 1.5 μg/μL and 10 μL of peptide solution was mixed with 90 μL 0.1M sodium acetate buffer pH 6.0 followed by the addition of 20 μL 4% formaldehyde-d_0_ (samples from the non-infected chickens) or formaldehyde-d_2_ (samples from the infected chickens) and 20 μL 1M NaBCNH_3_. After 10 min, the reactions were stopped by adding 20 μL 4% NH_3_ and the samples were randomly grouped into three pairs, each consisting of a sample from the infected and non-infected chicken.

LC-MS/MS analyses of dimethylated peptide mixture were done using RSLCnano system connected to Orbitrap Elite hybrid spectrometer (Thermo Fisher Scientific). Prior to LC separation, tryptic digests were concentrated and desalted using a trapping column (100 μm × 30 mm) filled with 3.5-μm X-Bridge BEH 130 C18 sorbent (Waters). After trapping the column was washed with 0.1% FA, the peptides were eluted (flow 300 nl/min) onto an Acclaim Pepmap100 C18 column (2 μm particles, 75 μm × 250 mm; Thermo Fisher Scientific) using the following gradient program (mobile phase A: 0.1% FA in water; mobile phase B: ACN:methanol:2,2,2-trifluoroethanol (6:3:1; v/v/v) containing 0.1% FA). The gradient elution started at 2% of mobile phase B and increased from 2% to 45% during the first 90 min, then increased linearly to 95% of mobile phase B in the next 5 min and remained at this state for the next 15 min. The analytical column outlet was directly connected to the Nanospray Flex Ion Source (Thermo Fisher Scientific).

MS data were acquired in a data-dependent strategy selecting the top 20 precursors based on the precursor abundance in the survey scan (350–1700 m/z). The resolution of the survey scan was 120 000 (at 400 m/z) with a target value of 1 × 10^6^ ions, one microscan and maximum injection time of 200 ms. Low resolution CID MS/MS spectra were acquired with a target value of 10 000 ions in rapid CID scan mode with the m/z range adjusted according to the actual precursor mass and charge. MS/MS acquisition in the linear ion trap was carried out in parallel with the survey scan in the Orbitrap analyzer by using the preview mode. Dynamic exclusion was enabled for 45 s after one MS/MS spectra acquisition and early expiration was disabled. The isolation window for MS/MS fragmentation was set to 2 m/z.

The analysis of the mass spectrometric RAW data files was carried out using the Proteome Discoverer software (version 1.3) with an in-house Mascot and Sequest search engine utilization. Mascot and Sequest MS/MS ion searches were performed against the NCBI protein database (taxonomy *Gallus gallus*). Mass tolerance for peptides and MS/MS fragments were 5 ppm and 0.5 Da, respectively. Carbamidomethylation (C), dimethylation (N-term and K) as fixed modification, oxidation (M) as optional modification and none enzyme miss cleavage were set for all searches. Percolator was used for post-processing of Mascot search results. Peptides with a false discovery rate (FDR; q-value) < 1% and at least 3 significant peptides were considered.

### Real-time PCR verification of the pyrosequencing data

Real-time PCR was used for the verification of both pyrosequencing and proteomic data. Primers for the quantification of expression real-time PCR were designed using Primer3 software [13] (for primer sequences see Additional file 1). First we used the same cDNAs as in the pyrosequencing reactions followed by an additional 2 × 4 samples from our previous study [6]. After such a screening we reduced the number of tested genes to those in which the real-time PCR confirmed the results from the pyrosequencing or proteomics and exhibited fold up- or downregulation greater than 4. Using the reduced set of real-time PCRs, we finally determined the gene expression in i) the time-dependent study and ii) after the infection with the SPI1 mutant.

Real-time PCR was performed in 3 μL volumes in 384-well microplates using QuantiTect SYBR Green PCR Master Mix (Qiagen) and a Nanodrop II Stage pipetting station (Innovadyne) for PCR mix dispensing. Real-time PCR was performed using a LightCycler II (Roche) with an initial denaturation at 95°C for 15 min followed by 40 cycles of 95°C for 20 s, 60°C for 30 s and 72°C for 30 s. Each sample was subjected to real-time PCR in duplicate and the mean Ct value of the duplicates was used for subsequent analysis. The Ct values of the genes of interest were normalized (ΔCt) to an average Ct value of three house-keeping genes (GAPDH, TBP and UB) and the relative expression of each gene of interest was calculated as 2^-ΔCt^. These expression levels were used for data analysis and are presented in the tables and figures as average ± SD. As an alternative, the fold upregulation (i.e. 2^-ΔΔCt^) calculated as the ratio of averages of infected to non-infected samples is presented. However, also in this case, the significance of such upregulations was calculated by comparing the expressions, i.e. the 2^-ΔCt^ values of the individual samples.

### Northern blot analysis

The expression of selected genes (MMP7, ExFABP, AQP8 and ADH1) was confirmed also by northern blotting. The same mRNAs of 6 ISA Brown chickens which was used for pyrosequencing was electrophoretically separated in formaldehyde denaturing agarose gel and vacuum blotted onto a nylon membrane as described previously [14]. Probes for the northern hybridization were prepared by PCR followed by purification using a PCR Purification kit from Qiagen according to the manufacturer’s instructions. Probe labeling, hybridization, posthybridization washes and signal development were performed with an AlkPhos labelling kit according to the manufacturer’s instructions (GE Healthcare). A GAPDH specific probe was used as a control of loading the same amount of RNA.

### Statistics

Heat maps were generated using software available elsewhere [15]. The statistical significance of the gene expression 4 days post infection were determined by a *t*-test. PCA have been calculated in logarithmically transformed data ignoring the absolute transcription levels and following the transcriptional profiles only. All the statistical calculations have been done using Statistica 9.1 software (StatSoft, Inc., Tulsa, OK, USA) and differences with *p* < 0.05 were considered as significant.

## Results

### Pyrosequencing

Pyrosequencing resulted in 96 459 reads (43 257 673 nt of RNA sequence) when pooled cDNA from the cecum of the non-infected chicken was sequenced and 43 458 reads (18 403 094 nt of RNA sequence) when pooled cDNA from the cecum of the chicken after *Salmonella* Enteritidis infection was sequenced. Combining these 2 samples together with the data sets from our previous study on the expression in chicken spleen [6], the *de novo* assembly resulted in the identification of 13 063 different chicken transcripts. After applying all the quality selective criteria, 35 457 reads from the cecum of the non-infected chicken and 29 586 reads from the cecum of the infected chicken were finally included in the quantification of gene expression and we predicted that 54 genes might be downregulated and 78 genes upregulated in the cecum after *Salmonella* Enteritidis infection (Additional file 1).

### Mass spectrometry

Using LC-MS/MS analysis, 4062 protein groups containing at least 1 peptide with a significant score were identified in an experimental set of 3 infected and 3 non-infected chicken samples. There were 806 protein groups which were quantified as having at least 3 peptides with a significant score and these were selected for further evaluation. At least a twofold down- or up-regulation was observed for 21 and 57 proteins, respectively (Additional file 1).

### Real-time PCR

In the next experiment we verified the expression of 195 genes predicted by either 454 pyrosequencing or mass spectrometry as responsive to *Salmonella* Enteritidis infection by real-time PCR. The predicted expression profiles were confirmed for 104 of them, with 48 genes confirmed as significantly downregulated and 56 as significantly upregulated (Additional file 1).

### Northern blot analysis

**Figure 1:**
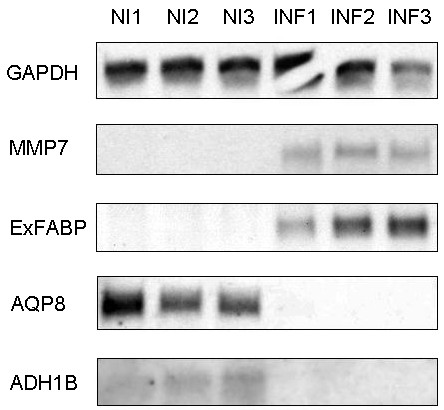
**Northern blot analysis for 4 selected genes responding to *Salmonella *Enteritidis infection in the cecum.** MMP7 and ExFABP were identified by real-time PCR as upregulated after the infection and AQP8 and ADH1 were identified as downregulated after the infection. GAPDH was used as a loading control. NI1, NI2 and NI3, mRNA purified from the cecum of non-infected chickens. INF1, INF2 and INF3, mRNA purified from the cecum of infected chickens.

Since all the above mentioned protocols can be considered as indirect, northern blot analysis was performed for 2 genes (AQP8, ADH1B) that were downregulated after infection and 2 genes (ExFABP, MMP7) that were upregulated after infection using samples originating from those used for pyrosequencing and mass spectrometry. The northern blot analysis confirmed the results of real-time PCR in all 4 cases (Figure 1).

### Time-dependent expression in the cecum of *Salmonella* Enteritidis infected chickens

**Figure 2:**
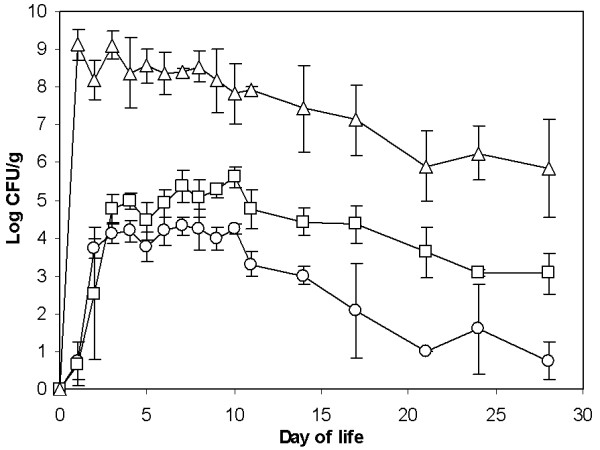
***Salmonella * Enteritidis colonization of the cecum, liver and spleen after oral infection at the day of hatching.** The cecum (triangles) was colonized as early as at the first sampling time, 24 h after the infection. Colonization of the liver (circles) and spleen (squares) required an additional 24 h and these organs became highly colonized from 48 h after the infection. The liver and spleen remained colonized at constant counts until day 12 of life and thereafter a gradual decrease in *Salmonella* Enteritidis counts was observed.

In previous experiments, gene expression was determined only at a single time point when the expression of some of the genes could have been already in decline whilst the expression of the others could still have been on the increase. In the next experiment we therefore characterized the chicken’s response to *Salmonella* Enteritidis infection in a time-dependent study. *Salmonella* Enteritidis counts in the cecum reached a maximum as early as 24 h after oral infection and then gradually decreased. The liver and spleen became highly colonized 48 h after oral infection. *Salmonella* Enteritidis counts in the liver and spleen then remained relatively constant until day 12, from which point on, a gradual decrease in *Salmonella* Enteritidis counts was observed (Figure 2).

**Figure 3:**
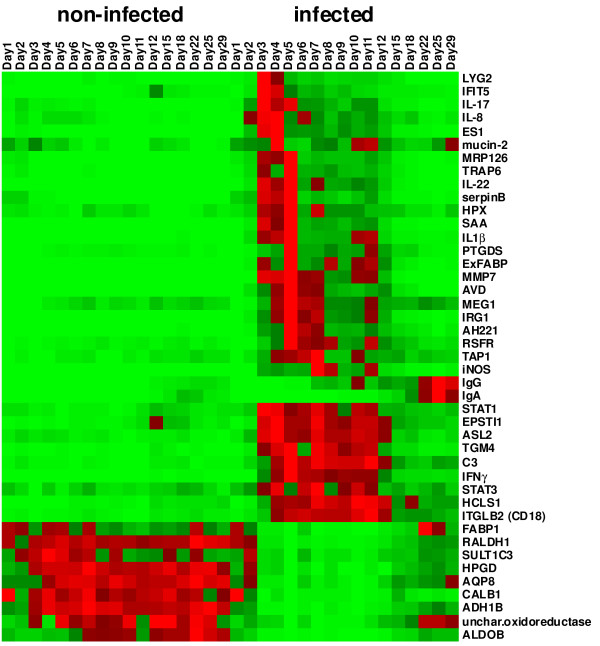
**Heat map of gene expression in the chicken cecum, with or without *Salmonella *Enteritidis infection.** The heat map for each gene was calculated based on its minimal and maximal recorded expressions. The color coding (light green, the lowest expression, light red, the highest expression) thus characterizes each gene profile and the same shade of red or green color for two different genes does not necessarily correspond to the same fold increase.

For monitoring time-dependent gene expression in the ceca we selected only 43 different genes. These included 32 genes identified in this study which were up- or down-regulated more than 4-fold. In addition, we also included 7 genes (avidin, trappin-6, chemokine AH221, IRG1, serum amyloid A, IgG, IgA) which we identified previously as responding to *Salmonella* Enteritidis infection [6] and 4 genes (IL-1β, IL-17, IL-22, IFNγ) which are commonly used as markers of inflammation [3-5]. There were 3 groups among the genes that showed induction after *Salmonella* Enteritidis infection which differed in the times of their maximal expression. The first group of genes was characterized by peak expression 2, 3 or 4 days after infection. This group included genes coding for lysozyme G2, IL-8, IL-17, serum amyloid A, trappin-6, ExFABP, ES1, IL-1β and prostaglandin synthase (Figure 2). The second group of genes exhibited a prolonged, high level expression and these included genes coding for STAT1, STAT3, epithelial stromal factor, arginine-succinate lyase, hematopoietic lineage protein, CD18, C3 complement protein and IFNγ. The last group of genes upregulated after *Salmonella* Enteritidis infection was formed by those coding for the constant part of the IgG and IgA heavy chains. Their expression increased above background levels for the first time on day 7 (IgG) and day 15 (IgA), respectively, and reached their maximal expression on day 22. Except for the immunoglobulin encoding genes, the expression of all the upregulated genes decreased between days 12 and 15 nearly to the levels observed in the non-infected chickens (Figure 3).

The genes which were downregulated in response to *Salmonella* Enteritidis infection exhibited such a profile from 48 h after the infection and their suppression lasted until day 15, from which time their expression began to recover (Figure 3).

### Contribution of individual genes to total expression in chicken cecum

Since the heat map in Figure 3 was normalized for each individual gene according to its minimal and maximal expression, it did not provide a realistic view of how individual genes contribute to the total gene expression in the cecum. Gene expression in the cecum of the non-infected chickens was dominated by aquaporin 8, alcoholdehydrogenase, fatty acid binding protein 1, retinal dehydrogenase, uncharacterized oxidoreductase and calbindin 1 (Table 1), i.e., the genes which were suppressed after *Salmonella* Enteritidis infection. Two days after infection, the above mentioned genes therefore formed a clear “expression” minority and they reached their minimal expression on day 5 when the transcription in the cecum was dominated by ES1 protein, avidin, AH221 chemokine, ExFABP and serum amyloid A (Table 1). In addition, high expression of IgG and IgA was recorded from day 15 reaching its maximum on day 22 and 25, respectively (Table 1).

**Table 1:** **List of genes up- or down-regulated in the chicken cecum after infection with *Salmonella *Enteritidis arranged according to their maximal fold up- or down-regulation after infection with wild type *Salmonella *Enteritidis (column max fold SE wt)**

**Gene**	**Max NI^#^**	**Min NI**	**Max SE**	**Min SE**	**Max fold**	**WT fold**	**SPI1 fold**
					**SE wt^$^**	**4 dpi**^**&**^	**4 dpi**^**&**^
MMP7	0.078 (12)	0.001 (29)	8.98 (5)	0.001 (1)	5952 (10)	1578	35.2
IgG	5.02 (25)	0.006 (2)	36.7 (25)	0.009 (1)	442 (10)	33.2	1.63
IRG1	0.005 (2)	0.001 (11)	0.69 (5)	0.003 (1)	425 (7)	222	4.61
SAA	0.25 (2)	0.018 (9)	15.9 (5)	0.065 (1)	382 (5)	106	5.02
ExFABP	1.06 (18)	0.150 (9)	75.3 (5)	0.369 (1)	366 (5)	56.8	4.80
IL-22	0.005 (12)	0.0004 (10)	0.39 (5)	0.001 (1)	355 (10)	46.9	2.69
TRAP6	0.39 (2)	0.011 (15)	5.76 (5)	0.029 (25)	194 (5)	37.3	2.19
MRP126	0.16 (2)	0.003 (29)	1.96 (5)	0.018 (25)	194 (11)	14.3	2.11
IFNγ	0.001 (18)	0.0001 (11)	0.035 (5)	0.0002 (1)	183 (11)	62.8	1.39
IL1β	0.018 (18)	0.002 (1)	0.74 (5)	0.002 (1)	134 (3)	29.1	1.59
iNOS	0.019 (1)	0.008 (9)	1.99 (7)	0.019 (1)	151 (7)	47.9	2.84
ES1	2.06 (2)	0.055 (29)	39.6 (4)	0.178 (1)	139 (11)	17.4	2.21
LYG2	0.26 (7)	0.015 (22)	5.82 (3)	0.031 (25)	130 (3)	24.5	3.09
IFIT5	0.56 (12)	0.003 (1)	1.28 (3)	0.003 (1)	124 (3)	42.4	2.62
IL-17	0.014 (2)	0.0003 (29)	0.095 (3)	0.001 (22)	120 (11)	6.72	1.12
AVD	2.07 (2)	0.20 (29)	103.1 (5)	0.207 (1)	116 (9)	29.6	3.24
AH221	0.73 (18)	0.12 (1)	23.4 (5)	0.119 (1)	108 (5)	42.9	1.90
serpin B	0.096 (2)	0.002 (29)	0.54 (5)	0.009 (25)	101 (5)	18.4	1.45
TGM4	0.092 (12)	0.005 (6)	0.92 (5)	0.014 (1)	68 (6)	30.4	1.99
IgA	6.04 (18)	0.003 (2)	23.4 (22)	0.005 (3)	49 (3)	16.6	1.86
EPSTI1	0.39 (12)	0.015 (1)	0.74 (4)	0.015 (1)	33 (3)	12.9	1.40
IL-8	0.15 (6)	0.004 (29)	1.08 (4)	0.007 (1)	31 (11)	4.54	1.24
C3	0.092 (2)	0.019 (10)	0.78 (11)	0.044 (1)	29 (10)	9.7	1.53
mucin-2	0.37 (3)	0.018 (9)	0.77 (4)	0.124 (6)	20 (11)	1.01	0.94
PTGDS	1.50 (9)	0.12 (1)	17.8 (5)	0.117 (1)	19 (5)	14.9	1.70
ITGLB2 (CD18)	0.084 (22)	0.031 (3)	0.46 (11)	0.045 (18)	14 (11)	9.40	1.04
HPX	0.19 (7)	0.025 (12)	0.91 (5)	0.071 (25)	13 (5)	5.06	1.10
ASL2	0.46 (2)	0.15 (29)	2.26 (4)	0.289 (1)	11 (10)	3.74	1.17
HCLS1	0.25 (29)	0.088 (5)	1.11 (10)	0.107 (1)	10 (11)	5.41	1.13
TAP1	0.443 (22)	0.087 (3)	2.26 (7)	0.104 (1)	10 (4)	13.7	1.37
STAT1	0.31 (12)	0.059 (9)	1.03 (3)	0.164 (1)	9.2 (9)	8.61	1.25
RSFR	0.96 (8)	0.40 (1)	7.19 (5)	0.397 (1)	9.1 (5)	6.41	1.19
MEG1	0.49 (22)	0.090 (3)	1.80 (5)	0.115 (1)	7.0 (5)	8.22	0.92
STAT3	0.64 (18)	0.37 (3)	1.28 (7)	0.537 (5)	2.2 (3)	2.91	1.00
AQP8	33.4 (22)	14.9 (1)	17.6 (25)	0.290 (5)	97 (5)	18.90	1.80
ADH1B	9.46 (7)	3.42 (2)	4.66 (25)	0.100 (5)	75 (5)	16.23	1.55
FABP1	6.11 (22)	1.98 (11)	8.48 (22)	0.119 (5)	47 (5)	5.49	1.32
RALDH1	18.6 (22)	9.17 (2)	12.6 (1)	0.404 (5)	39 (5)	12.87	1.43
unchar. oxidoreductase	5.84 (12)	0.80 (4)	4.25 (22)	0.192 (5)	28 (5)	7.14	1.26
CALB1	24.2 (1)	8.20 (2)	24.2 (1)	0.785 (5)	19 (5)	13.78	1.88
ALDOB	1.66 (22)	0.32 (2)	0.58 (22)	0.037 (5)	19 (10)	6.71	0.99
SULT1C3	21.9 (4)	7.20 (29)	16.0 (1)	1.081 (5)	18 (5)	8.04	1.86
HPGD	4.62 (7)	1.58 (1)	2.77 (2)	0.396 (5)	10 (7)	8.87	1.29

# Maximal or minimal expression levels observed in the non-infected or *Salmonella* Enteritidis infected chickens as determined in the time-dependent study; numbers in brackets indicate the day of life when the appropriate value was recorded.

$ Maximal fold upregulation or downregulation and the day when recorded in the time-dependent study. The downregulated genes are at the bottom of the table starting with AQP8.

& Fold up or downregulations 4 days post-infection of chickens with wild-type *Salmonella* Enteritidis and the SPI1 mutant.

### Identification of genes with the highest up- or down-regulation

The absolute expression of each of the 43 tested genes, however, need not correlate with their fold induction after *Salmonella* Enteritidis infection. The most downregulated gene was that coding for aquaporin 8 with a downregulation as low as 97 fold observed 5 days post-infection. The suppression of the remaining genes, though lower than that of aquaporin 8, were quite similar and ranged from 18 (SULT1C3) to 75 (ADH1B) fold (Table 1). The gene with the highest induction was that encoding matrix metalloproteinase MMP7 whose expression increased more than 3 logs with the highest 5952 fold upregulation observed 9 days after the infection. Upregulation of an additional 17 genes exceeded a factor of 100 and upregulation of the remaining 16 genes was below a factor of 100 (Table 1).

### PCA of individual chickens based on their gene expression in the cecum

**Figure 4:**
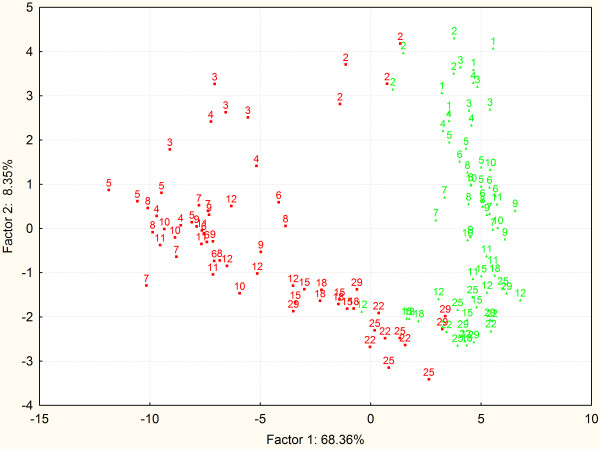
**PCA of the chickens clustered according to their gene expression in the cecum.** Sixty-eight percent of variability explained by coordinate 1 is likely to be associated with the infection status. Coordinate 2 explaining an additional 8% of variability seems to be chicken age. Green and red color, clustering of the non-infected and infected chickens, respectively. Numbers indicate age of each particular chicken in days.

The time-dependent experiment was performed with 132 chickens, samples of which were subjected to 43 gene characteristics which resulted in a dataset with 5940 values. This dataset was next used for the clustering of each individual chicken according to its expression profile. PCA clustering of the expression profiles observed in individual chickens confirmed and further extended previous analyses. The gene expression gradually changed in time also in the cecum of the non-infected chickens (Figure 4). *Salmonella* Enteritidis infection on the day of hatching resulted in a minor response in the proceeding 24 h and the 2-day-old infected chickens still clustered close to the non-infected age-matched controls. However, from 2 days post infection, the expression in the cecum clustered the infected chickens separately from the non-infected controls. PCA also indicated a gradual re-joining of the infected chickens with the non-infected ones from day 15 onwards and some of the infected chickens aged 22 days or more already merged with the non-infected controls (Figure 4).

### Influence of the SPI1-encoded type III secretion system of *Salmonella* Enteritidis on chicken gene expression in the cecum

*Salmonella* invasion of non-professional phagocytes is mediated by a type III secretion system encoded by Salmonella Pathogenicity Island 1 (SPI1) [16]. In the final experiment we therefore addressed to what extent the response in the chicken cecum to *Salmonella* Enteritidis infection depends on an intact SPI1. To test this hypothesis, we infected chickens with wild type *Salmonella* Enteritidis and an isogenic mutant in which the entire SPI-1 region was deleted [9]. Whilst the infection with the wild-type *Salmonella* Enteritidis induced expression to a similar extent as in the time-dependent experiment, the SPI1 mutant essentially did not induce an immune response in the chicken cecum (Table 1). Only MMP7 was significantly induced in the cecum 4 days after the infection with the *Salmonella* Enteritidis SPI1 mutant. However, the fold induction (35 fold) after infection with the SPI1 mutant was 45× lower when compared with the MMP7 induction in the cecum of chickens infected with the wild-type *Salmonella* Enteritidis (1578 fold, see Table 1). This difference could not be caused by a mere absence of the SPI1 mutant from the chicken cecum, liver or spleen, since the SPI1 mutant was detected in all of these tissues, though at lower counts than the wild-type *Salmonella* Enteritidis (Table 2).

**Table 2:** **Wild-type *Salmonella *Enteritidis and the SPI1 mutant colonization of liver, spleen and cecum 4 days after the infection**

	**Liver (CFU/g)**	**Spleen (CFU/g)**	**Cecum (CFU/g)**
*Salmonella* Enteritidis	4.13 ± 0.70	4.31 ± 1.80	8.41 ± 0.57
*Salmonella* Enteritidis ΔSPI1	2.95 ± 1.58	2.19 ± 1.71	7.78 ± 0.59

## Discussion

In this study we determined gene expression in the chicken cecum after *Salmonella* Enteritidis infection. Out of 9 genes downregulated after *Salmonella* Enteritidis infection expression of which we characterised in detail, 15-hydroxyprostaglandin dehydrogenase (HPGD) might be directly involved in host defense. 15-hydroxyprostaglandin dehydrogenase is an enzyme involved in the inactivation of prostaglandin D2. Interestingly, among the genes with increased expression, prostaglandin D2 synthase (PTGDS) has been identified. An increase in the expression of an enzyme required for the biosynthesis of prostaglandin D2 together with a decreased expression of the inactivating enzyme suggest that prostaglandin D2 accumulates in the cecum of infected chickens as one of the inflammatory signals.

Among the genes upregulated after the infection, induction rates greater than 100 fold were observed for MMP7, IgG, IRG1, SAA, ExFABP, IL-22, TRAP6, MRP126, IFNγ, iNOS, ES1, IL-1β, LYG2, IFIT5, IL-17, AVD, AH221 and SERPIN B. On the other hand, genes which exhibited the highest expression levels, irrespective of their fold increase, included AVD, ExFABP, ES1, IgG, IgA, AH221, PTGDS, SAA, MMP7, RSFR, LYG2 and TRAP6. Comparison of these two sets of genes showed that highly inducible cytokine encoding genes were absent among the genes with the highest absolute level of expression. This points to one limitation of our study. Since we purified total mRNA and/or protein from total cecal tissue, we could not detect changes in the gene expressions which may have occurred in the cells forming a minority in the gut tissue even if the change in expression levels within specific cells reached a factor of 100, as was the case of IL-17, IL-22 and IFNγ expressed by T-lymphocytes [17].

Although the majority of the genes identified in this study have not been associated with the chicken response to *Salmonella* Enteritidis infection, they have been described as inducible during different inflammatory diseases, cell differentiation or cancer. Based on the composition of genes upregulated after the infection it is also clear that the response was a coordinated action of epithelial cells (induction of MMP7, LYG2, IL-8), T-lymphocytes (IL-1β, IL-17, IL-22, IFNγ), macrophages and heterophils (IRG1, SAA, ExFABP, TRAP6, MRP126, iNOS, AVD, AH221, IFIT5) and B-lymphocytes (IgG, IgA).

Except for MMP7, the gene expressions characterized in this study were not triggered after infection with the non-invasive SPI1 mutant of *Salmonella* Enteritidis. However, even in the case of MMP7, its induction 4 days post-infection with the SPI1 mutant (35 fold) was significantly lower than after infection with wild-type *Salmonella* Enteritidis (1578 fold). This means that the signaling was strictly dependent on *Salmonella* invasion into non-professional phagocytes encoded by SPI1 [16] and the intracellular presence of *Salmonella* Enteritidis.

Two genes upregulated after infection exhibited quite a different pattern of expression from the “inflammatory” ones and these included the transcripts coding for the constant parts of IgG and IgA. As we proposed previously, the level of transcription of IgG and IgA increases in the chicken cecum with age even in the non-infected chickens [6] and infection of chickens with *Salmonella* Enteritidis increased the expression of IgG and IgA further. Maximal IgG and IgA expression after the infection was recorded when the expression of the remaining inflammatory genes began to decline. Interestingly, Wei et al. reported that the interaction of B-lymphocytes and CD8 T-lymphocytes was necessary for a decrease of CD4 T-lymphocyte activity at the site of epithelial inflammation and this interaction was dependent on TAP1 [18,19], another gene upregulated during the infection but declining when IgG and IgA increased in expression. TAP1 is involved in the presentation of self antigens in association with MHCI and increased expression of TAP1 was recorded in piglets in response to colonization with conventional microbiota [20], or in the lungs of *Salmonella* infected pigs [21]. This would point towards an important role of immunoglobulins or B-lymphocytes in the final recovery phase of infection. Another gene which was induced in the chicken cecum and may support the hypothesis on an important role of immunoglobulins or B-lymphocytes is HCLS1. HCLS1, also known as HS1, is expressed in all haematopoetic cells [22] and is involved in antigen-receptor signaling in both T- and B-lymphocytes. After cross-linking of the B-cell receptor, HCLS1 is phosphorylated [23] and associates with the B-cell receptor in lipid rafts of murine and chicken B-lymphoid tissue cultures [24]. Moreover, HCLS1 knockout mice are less responsive to T-cell independent antigens [25]. This suggests that the recovery phase is associated with B-lymphocytes and immunoglobulin production against T-cell independent antigens such as LPS. This is completely consistent with the use of LPS ELISA for the detection of anti-Salmonella antibodies [8]. Moreover, two groups of Crohn’s disease patients have been reported, either with increased or decreased B1-like cells producing low affinity antibodies in a T-cell independent manner. Interestingly, patients with decreased B1-like cells were subjected to surgery more frequently than the patients with increased B1-like cells in the intestinal tract [26]. This further supports the hypothesis that the recovery phase is dependent on immunoglobulin production and that these antibodies might be targeted primarily against thymus independent antigens such as *Salmonella* LPS [8].

Based on our results we propose that the chicken immune response to *Salmonella* Enteritidis infection occurs in four steps. During the first step IL-8 and IL-17 signaling is triggered and the simplest defense mechanisms involving lysozyme G2 and type I interferon inducible protein IFIT5 are activated. This phase peaks at 2 days post-infection. The second phase is associated with the expression of genes characteristic of heterophils and macrophages, and acute phase proteins such as IRG1, SAA, ExFABP, TRAP6, MRP126, iNOS, IL-1β, AVD or AH221. The peak of the phase 2 response is around day 4 post-infection. Between phase 1 and phase 2, MMP7 is induced allowing tissue relaxation and leukocyte influx and translocation across the intestinal epithelium. During the third phase from day 5 until day 15 post-infection, transcriptional regulators STAT1 and STAT3 are highly expressed, together with IFNγ characteristic of CD4 T-lymphocyte activity. In addition, iNOS, ASL, C3 complement, EPSTI1, HCSL1 and ITGB2 (CD18) were also induced. Expression of these genes is characteristic of the development of Th1 type of immune response and control of *Salmonella* Enteritidis infection. For the last phase, the expression of immunoglobulins is the most characteristic and the immunoglobulins are likely targeted against T-cell independent antigens. This is in total agreement with the known role of IgA as a major effector in the mucosal immune system [27].

## Competing interests

The authors declare that they have no competing interests.

## Authors’ contributions

MM and IR designed experiments and wrote the manuscript. MM was responsible for pyrosequencing. KV performed real time PCRs. KS and ZZ did the mass spectrometry. HH and FS performed animal experiments. VB was responsible for statistical analysis. All authors read and approved the final manuscript.

## Supplementary Material

Additional file 1 **List of differentially expressed genes identified in this study.** This Excel file contains additional information on differentially expressed genes identified in this study by 454 pyrosequencing, LC MS and real time PCR. In addition, primers used for the real time PCR are listed in this additional file as well. Click here for file
